# Association between route of furosemide administration and diuretic response in very preterm infants with bronchopulmonary dysplasia

**DOI:** 10.21203/rs.3.rs-8197255/v1

**Published:** 2025-12-01

**Authors:** Nicolas Bamat, Matthew Huber, Heidi Morris, Timothy Nelin, Kevin Downes, Anna O’Brien, Benjamin Laskin, Erik Jensen, Sara DeMauro, Eric Eichenwald, Scott Lorch

**Affiliations:** Children’s Hospital of Philadelphia; Children’s Hospital of Philadelphia; Children’s Hospital of Philadelphia; Children’s Hospital of Philadelphia; Children’s Hospital of Philadelphia; Children’s Hospital of Philadelphia; Stanford University School of Medicine and Lucile Packard Children’s Hospital Stanford; Children’s Hospital of Philadelphia; Children’s Hospital of Philadelphia

## Abstract

**Objective:**

Furosemide is commonly prescribed in hospitalized infants with grade 2–3 bronchopulmonary dysplasia (BPD). Intravenous (IV), gastric, and duodenal administrations are common, with a 1:2 IV-to-enteral conversion often used despite uncertain bioavailability. Our objective was to compare diuretic responses between routes in infants with BPD.

**Study Design:**

Single-center observational cohort of very preterm infants with grade 2–3 BPD prescribed furosemide. The association between route (exposure) and diuretic response (change in net fluid balance after administration, outcome) was evaluated using multivariable regression adjusting for dosing and infant characteristics.

**Results:**

Among 153 infants (median postmenstrual age of 43.3 weeks at exposure), furosemide reduced fluid balance by −25.6 (29.8) ml/kg/d. Adjusted mean changes were similar across routes: IV, −25.3 (−35.8, −14.7), gastric, −25.8 (−32.2, −19.4), and duodenal, −25.8 (−34.2, −17.4).

**Conclusions:**

Our data suggest a 1:2 IV-to-enteralconversion leads to comparable diuretic effects in infants with established BPD, supporting this common clinical practice.

## Introduction

Furosemide is the most frequently prescribed pharmacotherapy in infants with established bronchopulmonary dysplasia (BPD) admitted to neonatal intensive care units (NICU) in United States children’s hospitals.^1^ In a report of medication prescriptions among 3252 infants with BPD between 36 weeks postmenstrual age (PMA) and discharge, furosemide was prescribed at least once in 74% of infants and present in one-third of all patient-days across the study cohort.^1^

Despite its common use, clinical research evidence supporting furosemide use in BPD is lacking, and key knowledge gaps in its neonatal pharmacology remain. For example, furosemide’s enteral bioavailability – the proportion of drug reaching the circulation in active form - remains uncertain, with limited data from 4 preterm infants in the 1980s reporting a wide range (20–106%).^2,3^ Adult studies report typical bioavailability values between 60–70% and similarly note high variability.^4,5^ A recently published neonatal trial applied a 1:2 intravenous-to-enteral conversion consistent with 50% bioavailability, yet a Food and Drug Administration label update based on unpublished pharmacokinetic (PK) data from the same trial describes a bioavailability estimate of 79%.^6,7^ Data from the Pediatrix NICU network identify 1 mg/kg is the predominant intravenous (IV) dose prescribed in very preterm infants and while 2 mg/kg is the most common enteral dose, 1 mg/kg is also used often, highlighting uncertainty and practice variation.^8^

In infants with BPD, uncertainty surrounding enteral furosemide dosing is further complicated by the common use of duodenal feedings as a lung protection strategy.^9,10^ At our center, single-lumen oro- or nasoduodenal tubes are often used concurrently for feedings and medications, despite the possibility for differential absorption when drugs are administered to the duodenum. In a small cross-over PK study in adult volunteers, duodenal administration increased furosemide bioavailability by 30%, yet gastric furosemide led to a 12% greater diuretic effect over 8 hours.^11^ One explanation for this discordance is that gastric administration leads to more gradual absorption, maintaining furosemide concentrations within an optimal therapeutic window for a longer duration, and enhancing diuretic “efficiency”, as described by prior investigators.^11,12^

The objective of the current study was to compare the diuretic response to furosemide delivered by differing routes of administration in very preterm infants with BPD. Given the possibility that enteral bioavailability may often exceed 50% and that gastric dosing may enhance diuretic efficiency, we hypothesized that gastric administration would produce a greater diuretic response than either IV or duodenal administrations.

## Methods

### Study Design, Data Source and Population

**Figure 1:**
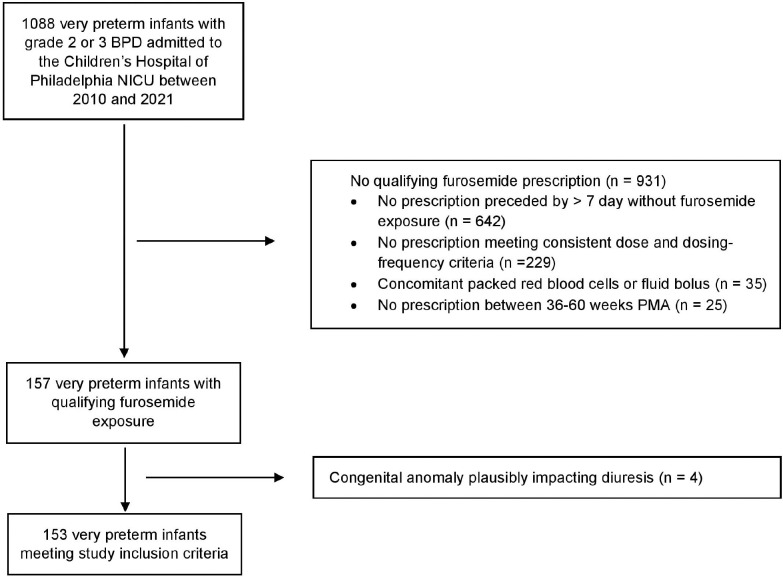
Flow diagram of the study population

We performed a single-center observational study using the clinical data warehouse at Children’s Hospital of Philadelphia (CHOP). This resource contains data extracted from the electronic health record (EHR), inclusive of time-stamped medication administration records and detailed, longitudinal flowsheet data input by bedside providers. Identification of the study population is shown in Fig. 1. We included very preterm infants (< 32 weeks birth gestational age) admitted to the CHOP NICU between 2010 and 2021 and classified with grade 2 or 3 BPD per the 2019 Neonatal Research Network/Jensen definition.^13^ BPD grade was based on the highest respiratory support used at 36 weeks PMA or at admission (when occurring after 36 weeks PMA). Furosemide prescriptions qualifying for study occurred between 36- and 60-weeks PMA, reflecting the age range of infants with established BPD routinely managed in our NICU. To avoid the influence of homeostatic tolerance on diuretic response, we excluded prescriptions that lacked at least 7 preceding days without furosemide exposure.^14,15^ Qualifying furosemide prescriptions were ordered as a one-time dose, or as repeated dosing with consistent route of administration and a milligram per kilogram (mg/kg) dose of 1 mg/kg IV or 1 or 2 mg/kg enteral every 12 or 24 hours. Single doses and repeated doses scheduled every 24-hours were grouped together as our primary outcome was based on diuretic response over the first 24 hours, a time-period over which these exposures are analogous. We excluded furosemide prescriptions with concomitant red blood cell or fluid bolus administrations to decrease excess variance in fluid balance measures. Lastly, we excluded infants with congenital anomalies that we deemed could plausibly impact diuretic responsiveness, identifying these by reviewing International Classification of Diseases diagnostic codes. The data used in this study were approved for collection under the CHOP Neonatal and Infant Chronic Lung Disease Program clinical data registry by the CHOP Institutional Review Board, #19–016420.

### Study Variables

The objective of our study was to determine the association between route of furosemide administration and diuretic response. The primary predictor variable was route of administration ascertained from the medication administration record and modeled as a nominal variable with 3 categories: IV, gastric, and duodenal. The primary outcome variable was diuretic response, defined as the within-subject change in total net fluid balance in the 24-hours before and after initiation of furosemide. Fluid balance was calculated as the net of all fluids administered minus all recorded outputs for each time interval. Input was inclusive of medication volumes, intravenous fluids, parenteral nutrition, and enteral feeding volumes, while output was inclusive of urine, stool, emesis and drains, when present. To adjust for inexact total time between repeated-dosage prescriptions, fluid balance was standardized as milliliters per kilogram per (24 hours) day (ml/kg/d), accounting for incomplete hours and days in the denominator.

To adjust for potential confounders between route of administration and diuretic response, we considered furosemide dosage and infant characteristics plausibly associated with diuretic response as covariates. Drug covariates were IV-equivalent dose and dose frequency, considered as separate variables. We reported all prescriptions as IV-equivalent doses, assuming a bioavailability of 50% for enteral formulations, such that a 2 mg/kg enteral dose was reported as 1 mg/kg IV-equivalent. These dosing characteristics reflect our typical institutional practice patterns. Under this approach, a similar diuretic response across route of administration would support our typical 1:2 IV to enteral conversion assumptions. Infant covariates were: birth gestational age group, in completed weeks; sex; BPD grade; PMA at furosemide exposure; plasma albumin, blood urea nitrogen (BUN), sodium and chloride levels, abstracted from the EHR at the time point nearest to furosemide exposure – prior to administration when available, or following exposure if not; estimated glomerular filtration rate using the Schwartz formula modified for preterm infants^16^; and co-administration of thiazides diuretics, hydrocortisone, dexamethasone, or dopamine, all categorized as absent or present on the day of exposure.

### Statistical Analyses

Cohort characteristics were summarized with descriptive statistics. The association between route of administration and diuretic response was modeled with linear regression, applying robust variance estimates. The unadjusted associations between route of administration and all candidate covariates were first examined in bivariable analyses. All characteristics associated with diuretic response at *p* < 0.10 were included with furosemide dose and frequency (selected *a priori*) as covariates in the multivariable model. In a *post-hoc* sensitivity analyses, the model was modified to include total daily furosemide dose (considering both administration dose and dose frequency; e.g 1 mg/kg/dose every 12 hours results in 2 mg/kg/day) rather than administration dose. *P*-values < 0.05 were considered statistically significant. Analyses were performed using STATA 17 (College Park, TX).

## Results

We identified 153 very preterm infants who met eligibility criteria (Figure 1). Characteristics of the full study cohort, and stratified by prescribed route of furosemide administration, are displayed in Table 1. Among eligible infants, 56% were born between 22 and 25 completed weeks GA and 67% had grade 3 BPD. The median (interquartile) PMA at furosemide exposure was 43.3 (39.6 – 47.9) weeks. Gastric was the most common route of furosemide administration (42%), followed by IV (32%), then duodenal (26%). The most common dose frequency was once or every 24 hours (69%). An IV-equivalent dose of 1 mg/kg was prescribed in 84% of infants, with the remaining 16% exposed to 0.5 mg/kg (equivalent to 1 mg/kg enteral furosemide). Among medication co-administrations, only thiazide diuretics were common (46%). The median laboratory values were largely within normal limits. All were obtained prior to furosemide exposure except 8 (6%) albumin values.

The mean (standard deviation) net fluid balances in the 24 hours before and after furosemide administration were +51.5 (23.2) and +25.8 (27.6) ml/kg/d, with a mean (standard deviation) diuretic response (within-subject change) of −25.6 (29.8) ml/kg/d across the full cohort. Table 2 displays the unadjusted bivariable association between evaluated characteristics and diuretic response. Diuretic response was similar across all routes of administration. Only dose frequency (greater diuretic response with every 12 vs every 24-hour dosing) and BUN (lower diuretic response with greater values) were statistically significant. Birth gestational age also met criteria for inclusion in the multivariable analysis.

Diuretic responses were similar across route of administration in adjusted multivariable modeling (Table 3), with estimated mean (95% confidence interval) values of −25.8 (−32.2, −19.4), −25.3 (−35.8, −14.7), and −25.8 (−34.2, −17.4) ml/kg for gastric, IV, and duodenal administrations, respectively. The effect estimates trended towards a greater diuretic response with more frequent and higher dosing but these associations lacked statistical significance. Greater BUN values were observed to have a statistically significant association with diuretic response, with higher values resulting in a smaller (less negative) diuretic response. The sensitivity analysis considering total daily furosemide dose rather than administration dose had consistent findings (Table 4).

## Discussion

The objective of our study was to compare the diuretic response to furosemide delivered by differing routes of administration in very preterm infants with BPD. We hypothesized that a gastric furosemide administration route would be associated with a greater diuretic effect than either an IV or duodenal administration when standardized to a 1:2 IV-to-enteral dose conversion. This hypothesis was based on the rationale that the enteral bioavailability of furosemide may exceed 50% on average and on prior research suggesting that gastric furosemide administration may be most “efficient” by sustaining therapeutic concentrations over a longer period.^12^ Our findings did not support our hypothesis. In the first 24 hours following administration, changes in net fluid balance were similar among the three routes. This pattern was consistent in both unadjusted bivariable analyses and adjusted models.

Opportunities to compare our findings with prior relevant research are limited. We are unaware of other neonatal studies evaluating how route of furosemide administration impacts diuretic response. Consistent with our findings, a pharmacokinetic study of 7 adult patients with congestive heart failure reported comparable urine output over 24 hours with IV vs oral furosemide.^5^ In turn, data from 4 healthy adult volunteers receiving gastric vs duodenal furosemide via endoscopy found 12% greater diuretic effect over 8 hours with gastric furosemide.^11^ While inconsistent with our findings, the relevance of these data is limited by the small sample size and differences in study population.

Our findings are relevant to the contemporary care of infants with BPD, in whom furosemide is the most frequently prescribed pharmacotherapy.^1^ Our data suggest that a 1:2 IV-to-enteral dose conversion leads to a comparable diuretic effect in infants with established BPD, lending support to this common, though variable, clinical practice.^6,8^ Furthermore, our findings provide reassurance that duodenal administration produces a comparable diuretic response in infants with BPD. Notably, no prior data exist on the effects of duodenal furosemide in preterm infants, despite its routine use in clinical practice.

Our models identified a statistically significant association between greater BUN levels and a lower diuretic response. This association is biologically plausible, as elevated BUN may reflect renal dysfunction – particularly reduced renal perfusion (i.e. pre-renal azotemia) – and may trigger neurohormonal pathways that limit urine output.^17^ Consistent with our finding, multiple clinical studies in adults with heart failure have reported an independent association between elevated BUN and a decreased diuretic response.^18,19^ However, our observation was *post hoc*, and replication in dedicated studies is needed to establish whether BUN could serve as a useful predictor of diuretic responsiveness in preterm infants.

Our study has several limitations. We used changes in net fluid balance as a surrogate measure of diuresis, relying on real-world clinical data. Although urine output and natriuresis are more direct measures of furosemide renal responsiveness, fluid balance is routinely assessed in clinical care and offers a pragmatic alternative, as intake strongly influences output and urine volumes cannot be reliably discriminated from stool in routine neonatal practice. Prior studies have similarly used net fluid balance as a marker of diuresis.^14,20–22^ Our finding of a 50% reduction in net fluid balance after furosemide administration reflects expected patterns, and supports the construct validity of this measure, which has also been demonstrated in prior studies.^14,20–22^ Our data are susceptible to information bias, such as erroneous route of administration assignment in the medication administration record, inaccurate measurements or calculations of fluid intake and output, or transcription errors into the electronic health record. Carefully conducted prospective studies may reduce these. Despite including 153 infants, our study lacked sufficient power to detect clinically meaningful associations. We expected both dose and dosing frequency would influence diuretic response and included them *a priori* in our models. Although effect estimates suggested a 52% greater response with every 12 vs 24-hour dosing and a 36% greater response with a 1 vs 0.5 mg/kg IV-equivalent dose, these sizeable differences did not reach statistical significance. Multicenter studies with larger sample sizes may allow greater statistical precision. Lastly, the generalizability of our findings is limited by our single-center design and by our focus on infants with established grade 2–3 BPD who received furosemide at a median PMA of 43 weeks. Prior studies suggests that furosemide pharmacokinetics, particularly renal clearance, change rapidly with maturation in preterm infants.^2^ Thus, our results may not extend to younger preterm infants.

In summary, our findings suggest that IV, gastric, and duodenal furosemide, when dosed using a 1:2 IV-to-enteral conversion, produce a similar initial diuretic response in preterm infants with established BPD, supporting the appropriateness of these dosing practices. Our findings do not necessarily imply a typical 50% bioavailability for gastric and duodenal furosemide, as bioavailability reflects drug exposure rather than clinical response. Characterization of the dose-exposure-response relationship requires further study, such as comparative bioavailability trials measuring circulating furosemide concentrations alongside diuretic effect. Finally, although furosemide is widely used in this population, its broader safety and effectiveness in improving meaningful outcomes in infants with BPD remain uncertain. Our findings will help inform the careful design of future phase III trials, which remain greatly needed.

## Supplementary Material

Supplementary Files

This is a list of supplementary files associated with this preprint. Click to download.

• Table1.docx

• Table2.docx

• Table3.docx

• Table4.docx

Tables 1 to 4 are available in the Supplementary Files section

## Abbreviations

BPD: bronchopulmonary dysplasia
NICU: neonatal intensive care unit
PMA: postmenstrual age
PK: pharmacokinetic
IV: intravenous
CHOP: Children’s Hospital of Philadelphia
EHR: electronic health record
mg/kg: milligram per kilogram
ml/kg/d: milliliters per kilogram per day
BUN: blood urea nitrogen
